# Recent Insights Into the Role of Immune Cells in Alcoholic Liver Disease

**DOI:** 10.3389/fimmu.2019.01328

**Published:** 2019-06-12

**Authors:** Sha Li, Hor-Yue Tan, Ning Wang, Yigang Feng, Xuanbin Wang, Yibin Feng

**Affiliations:** ^1^Li Ka Shing Faculty of Medicine, School of Chinese Medicine, The University of Hong Kong, Hong Kong, China; ^2^Guanghua School of Stomatology, Sun Yat-sen University, Guangzhou, China; ^3^Laboratory of Chinese Herbal Pharmacology, Laboratory of Wudang Local Chinese Medicine Research, Oncology Center, Renmin Hospital, Hubei University of Medicine, Shiyan, China

**Keywords:** immune cells, inflammation, alcoholic liver disease (ALD), innate immunity, adaptive immunity

## Abstract

Accumulating clinical and experimental evidences have demonstrated that both innate and adaptive immunity are involved in the pathogenesis of alcoholic liver disease (ALD), in which the role of immunity is to fuel the inflammation and to drive the progression of ALD. Various immune cells are implicated in the pathogenesis of ALD. The activation of innate immune cells induced by alcohol and adaptive immune response triggered by oxidative modification of hepatic constituents facilitate the persistent hepatic inflammation. Meanwhile, the suppressed antigen-presenting capability of various innate immune cells and impaired function of T cells may consequently lead to an increased risk of infection in the patients with advanced ALD. In this review, we summarized the significant recent findings of immune cells participating in ALD. The pathways and molecules involved in the regulation of specific immune cells, and novel mediators protecting the liver from alcoholic injury via affecting these cells are particularly highlighted. This review aims to update the knowledge about immunity in the pathogenesis of ALD, which may facilitate to enhancement of currently available interventions for ALD treatment.

## Introduction

**Figure 1:**
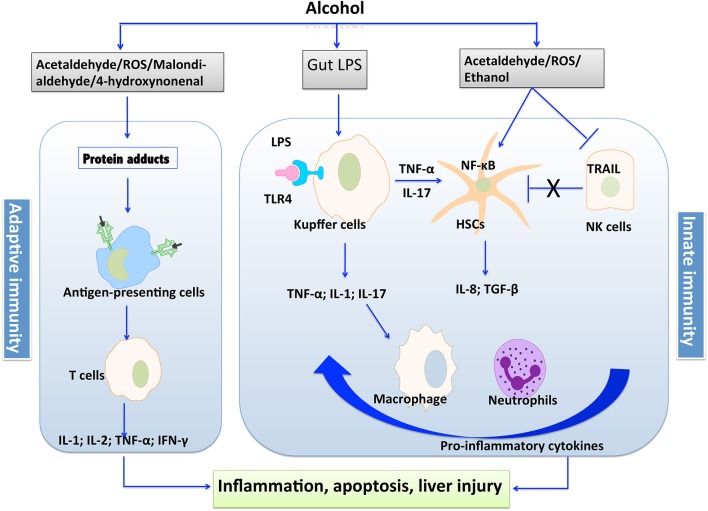
The process of immunologic priming induced by alcohol.

Alcoholic liver disease (ALD) encompasses a broad spectrum of liver injuries ranging from steatosis with a minimal parenchymal injury to steatohepatitis, fibrosis, and finally cirrhosis. Although there is limited progress in the therapy of ALD in recent years, plenty of studies have advanced our knowledge about the pathological process of liver injury induced by alcohol. Notably, the implication of immunity in fueling the inflammation and progression of ALD has been emerged by the accumulating clinical and experimental studies (1–3). It is well-known that both innate and adaptive immunity are involved in the pathogenesis of ALD. The leakage of microbes and microbial products from the damaged gastrointestinal tract, alcohol *per se*, and its intermediate metabolites are immune challenges to disturb the fine-tuned immune pathways in the liver (4, 5). A variety of cellular sensors of pathogen- or damage-associated molecular patterns (PAMPs/DAMPs) are further activated, resulting in the production of pro-inflammatory cytokines such as TNF-α and IL-1β, which leads to cellular dysfunction that contributes to ALD (5). The intermediate metabolites such as acetaldehyde and malondialdehyde trigger protein adducts, which could be recognized by antigen-presenting cells, leading to the response of adaptive immunity and production of pro-inflammatory cytokines. The process of immunologic priming induced by alcohol is shown in Figure 1.

Increasing evidences indicated that disturbed immunity is implicated in the development of ALD from early to an advanced stage. At the initial stage of ALD, impaired barrier function of the intestinal mucosa caused by alcohol leads to increased lipopolysaccharide (LPS) to the portal circulation, which further activates innate immunity via Toll-like receptors (TLRs) expressed by various immune cells, resulting in inflammatory response (6, 7). The induced pro-inflammatory cytokines, such as chemoattractant protein-1 (MCP-1), are involved in the early alcoholic hepatic steatosis. Subsequent alcohol abuse leads to the development of alcoholic hepatitis and fibrosis (6). At this stage, the oxidative breakdown of alcohol, acetaldehyde, constrains the function of certain immune cells such as natural killer (NK) cells that induce apoptosis of activated hepatic stellate cells (HSCs) to moderate fibrosis. Cytokines produced by inflammatory macrophages recruited from the periphery and Kupffer cells activate quiescent HSCs, leading to the proliferation of myofibroblasts that produce extracellular matrix proteins with the consequence of the generation of scar tissue (8). Moreover, CD8^+^ T-lymphocytes also contribute to the pro-fibrogenic activation of HSCs (9). About 10–20 percent of fibrosis patients without abstinence from alcohol might progress to cirrhosis, the final stage of ALD, which is characterized by fibrotic deformation of tissues and blood vessels, as well as necrosis of cells. The failure in liver's function to remove microbial and other circulating pro-inflammatory metabolites, as well as release of immunogenic cellular debris from necrotic hepatocytes ultimately cause persistent activation of immune pathways and aggravates the illness (10, 11).

Given the vital role of immunity in the pathological process of ALD, interventions targeting the immune system might represent the promising therapeutic approach for the treatment of ALD. The key components of the immune system are immune cells. Recent extensive studies into the roles of various immune cells in ALD have facilitated our understanding of the molecular basis of this disease. In this review, we summarize the major recent findings of participating immune cells in ALD. The pathways and molecules involved in the regulation of specific immune cells including innate and adaptive immune cells, and novel targets protecting the liver from alcoholic injury via affecting these multiple cell types are particularly highlighted.

## Innate Immune Cells

The cells of the innate immune system include macrophages, neutrophils, dendritic cells, NK cells, and NKT cells. On the one hand, they orchestrate innate immune response, which is the primary response to danger signals raised from stimulus or damaged host cells and tissues. On the other hand, they initiate adaptive immune responses via cell interactions, chemokines, cytokines, and other mediators secretion (12). Generally, inflammation is considered to be the most robust and typical response of the innate immune system to danger signals. Alcohol-induced activation of innate immunity in liver precipitates disorders ranging from localized and temporary inflammation to extensive hepatocellular damage and tissue injury (13, 14).

### Macrophages and Kupffer Cells

#### Macrophage Activation and Recruitment

The recruitment of macrophages and activation of Kupffer cells, a liver resident macrophage subpopulation, are critical cellular events of ALD pathogenesis. Generally, the increased intestinal permeability and injured hepatocytes induced by alcohol consumption lead to the release of pathogen- or damage-associated molecular patterns (PAMPs/DAMPs). Then these molecular patterns activate Kupffer cell through TLRs pathway (15–17), thereby leading to hepatic inflammation and exacerbating ALD. The plasma level of soluble CD163, a specific marker of inflammatory macrophage activation, was increased in patients with stable alcoholic cirrhosis compared with that in healthy individuals (18). It was considered as an independent predictor of the 84-day mortality of alcoholic cirrhosis patients. The contents of several critical components of the LPS pathway including plasma LPS, soluble CD14, and LPS-binding protein were increased to the same degree as soluble CD163 in these patients, suggesting that the activation of hepatic macrophage possibly via LPS pathway (18).

Infiltrating macrophages, which are differentiated from circulating monocytes in the liver, have several subtypes with opposite functions. Macrophages derived from Ly6C^low^ infiltrating monocytes showed an anti-inflammatory and protective phenotype while Ly6C^high^ infiltrating monocytes present a pro-inflammatory and tissue detrimental phenotype. The ratio of Ly6C^high^/Ly6C^low^ increases when mice exposed to ethanol, leading to significant liver injury. However, pro-inflammatory Ly6C^high^ monocytes could switch to anti-inflammatory Ly6C^low^ cells upon phagocytosis of apoptotic hepatocytes, suggesting the complex role of macrophages in the pathogenesis of ALD (19).

#### Macrophage Polarization

Increasing evidences showed that different macrophage subpopulations exhibit diverse effects in the context of ALD. Macrophages have a unique capacity to change their phenotypes and functions depending on surrounding factors, such as cytokines and growth factors (20). There are two major macrophage phenotypes: M1 and M2 macrophages. The M1 macrophage, which are activated by TLRs agonists, PAMPs and DAMPs, produce inflammatory cytokines and chemokine such as TNF-α, IL-1β, CCL2, and CXCL1, and result in tissue inflammation and damage (20). When the injury is controlled, M1 might switch to an anti-inflammatory and tissue-repairing phenotype, the alternatively activated macrophages (M2). The M2 macrophage is generally activated by IL-4/IL-13 and dead cells, and these macrophages resolve inflammation via production of anti-inflammatory mediators such as IL-10 (21). Meanwhile, macrophage expressing markers TLR-2, TLR-4, and TLR-8 as well as the chemokine CCL-1 and CCL-18, were detected in both M1 and M2, indicating that a complex interplay between different types of macrophages is implicated in alcoholic hepatitis (22). As shown by liver biopsies from patients with alcoholic hepatitis, robust overexpression of TGF-β, a growth factor associated with fibrogenic properties in M2 macrophages was observed.

The role of telomerase reverse transcriptase (23) in macrophage regulation of ALD has been demonstrated by a recent research (24). The TERT expression and telomerase activity were significantly increased in liver tissue of mice receiving ethanol treatment. *In vitro* study showed that ethanol could up-regulate the expression of TERT in Kupffer cells and RAW 264.7 cells. It was shown that TERT switched macrophages toward M1 phenotype via regulation of the NF-κB signaling pathway, while showed a limited effect on M2 macrophages polarization. Furthermore, TERT expression and M1 macrophage hallmarks were significantly reduced by NF-κB inhibitor, suggesting the cross-talk between TERT and p65. TERT might be partially responsible for ethanol-mediated hepatic inflammation response and M1 macrophage polarization (24). In another study, Kruppel-like factor 4 (KLF4) has also been identified as a key mediator of M1/M2 macrophage polarization in ALD. Ethanol promotes the induction of KLF4 and M2 phenotype, whereas acetaldehyde diminishes KLF4 and facilitates M1 macrophage, which may elucidate the increased populations of M1 and M2 macrophage in ALD (25).

#### Emerging Mediators

Emerging studies have identified a variety of mediators that regulate the activation and polarization of macrophage in response to alcohol. Targeting these mediators might be an effective intervention for treating ALD. Some of these factors positively promoted the activation and polarization of macrophages toward inflammatory phenotype via NF-κB signaling, which further drives the process of alcoholic liver injury. Macrophage migration inhibitory factor (26), a multipotent cytokine that contributes to the inflammatory response to injury, plays a critical role in the pathogenesis of ALD in mice and patients (27). The serum contents of MIF in patients with alcoholic-related liver hepatitis and cirrhosis were higher than healthy controls and positively correlated with the serum transaminase levels (28). In the liver of alcohol-treated MIF-/- mice, the expression of TNF-α was attenuated due to reduced F4/80+ macrophages population. Moreover, chronic alcohol feeding failed to sensitize MIF^−/−^ mice to LPS, leading to the decreased chemokine production and monocyte recruitment into the liver (29). These studies evidenced that MIF is an essential mediator in the regulation of chemokine expression and immune cell infiltration in the liver during the ethanol-induced liver injury (27–29).

Increasing number of studies showed that iron accumulation in macrophage that associated with NF-κB activation is a crucial feature of ALD (24). Chronic alcohol administration increased expression of transferrin receptor-1 and hemochromatosis gene, enhanced iron uptake, and accentuated intracellular labile iron response for NF-κB activation in Kupffer cells, resulting in significant TNF-α production. The enhanced iron uptake is responsible for iron loading in Kupffer cells, and the intracellular labile iron response is a function acquired by differentiated macrophages in humans, serving as a priming mechanism for alcoholic liver injury (30). A variety of micro-RNAs (miR) such as miR-125b, miR-146a, and miR-155 are involved in inflammatory responses to LPS. In the case of chronic alcohol treatment, miR-155 was increased in RAW 264.7 macrophages via the NF-κB pathway (31). The increased miR-155 further facilitates alcohol-induced production of TNF-α via enhancing mRNA stability (31). Ethanol can synergize with LPS to induce TNF-α by reducing the cellular cAMP levels in monocytes/macrophages, indicating that cAMP-elevating agents might be a practical therapeutic approach in counteracting the progression of ALD (32). Moreover, extracellular vesicles, which could transfer biomaterials such as proteins and microRNAs and serve as important effectors of intercellular communication, have been demonstrated to modulate the Kupffer cell phenotype and result in inflammatory activation in the setting of alcoholic liver injury. Extracellular vesicles mediated the increased percentage of TNF-α^+^ IL-12/23^+^ M1 Kupffer cells and decreased the population of CD206^+^CD163^+^ M2 Kupffer cells in mice with ALD. Furthermore, the increased of heat shock protein 90 (hsp90) in circulating extracellular vesicles of alcoholic mice was found to contribute to the activation of macrophage (33). The inhibition of hsp90 could reduce inflammatory cells, decrease sensitization of hepatic macrophage to LPS, leading to NF-κB inhibition and diminished pro-inflammatory cytokine production in mice with alcoholic liver injury (34).

On the other hand, a series of mediators, such as Cannabinoid CB2 receptors, adiponectin, IL-10 and IL-1 receptor like 1, have been shown to attenuate alcoholic liver injury via acting on macrophages (35, 36). Cannabinoid CB2 receptors are G protein-coupled receptors, which are mainly expressed by immune cells such as macrophages. They exert protective effects on ethanol-induced liver injury via regulating M1/M2 balance in Kupffer cells. CB2 receptors inhibited M1 polarization and promoted the transition to an M2 phenotype, thereby diminishing hepatocyte steatosis via paracrine interactions between hepatocytes and Kupffer cells (37). Further mechanism research revealed that CB2 receptor inhibited alcohol-induced hepatic inflammation in an autophagy-dependent manner via hemeoxygenase-1 (HO-1) induction (38). The CB2 receptor agonist-induced macrophage autophagy to reduce hepatic steatosis in wild-type mice after chronic-plus-binge alcohol feeding, but not in mice invalidated for autophagy gene ATG5 in the myeloid lineage, suggesting that the anti-inflammation and anti-steatogenic effects of the CB2 receptor is mediated by macrophage autophagy (38).

### Neutrophil

The infiltration of neutrophils into liver is a prominent feature of ALD (39, 40). Overwhelming evidence from clinical and animal model studies has shown that alcohol-induced neutrophil infiltration in liver contributes to the pathological manifestations of ALD (40), possibly through production of ROS and proteases to kill hepatocytes. Human neutrophil peptide (HNP)-1, an antimicrobial peptide secreted by neutrophils, exacerbated alcohol-induced liver fibrosis and hepatocyte apoptosis via down-regulating Bcl2 expression and up-regulating miR-34a-5p expression (41). The mechanisms by which neutrophils were recruited to the liver during alcoholic liver injury have been extensively studied. Neutrophil recruitment requires the interaction of multiple adhesion molecules and their ligands that are expressed by endothelial cells and neutrophils, such as E-selectin and P-selectin, vascular cell adhesion molecule 1 (VCAM-1) and intercellular adhesion molecule 1 (ICAM-1). A study has demonstrated that ICAM-1 deficiency significantly reduced neutrophil infiltration and alleviated liver injury in a mouse model with continuous enteral alcohol treatment (42). In another study, genetic disruption of E-selectin eradicated neutrophil infiltration, liver injury, and inflammation induced by chronic-binge ethanol treatment, suggesting its pivotal role in the pathogenesis of ALD at the early stage (43).

Increasing number of mediators have been demonstrated to be involved in neutrophils infiltration and pathogenesis of alcoholic liver injury by recent studies. In patients with severe alcoholic hepatitis, a significant increase in albumin oxidation was observed, which plays a role in neutrophils activation, and subsequently induced hepatic oxidative stress and inflammation (44). Plasma level and hepatic expression of CCL2 were also found to significantly increase in alcoholic hepatitis patients and were associated with the severity of the disease. The hepatic expression of CCL2 was correlated with neutrophil infiltration and IL-8 expression, indicating that CCL2 might be implicated in the pathogenesis of ALD via neutrophil recruitment (45). Moreover, hepatocytes and HSCs generate high amounts of CXCL1 through TLR2 and TLR9-dependent MyD88 pathway, which then stimulates neutrophil infiltration into the liver via CXCR2, leading to the progression of alcohol-mediated liver injury (46). Lipocalin-2, a siderophore binding peptide, has been demonstrated to drive ethanol-caused neutrophilic inflammation and contribute to the development of ALD (47). Recently, it was demonstrated that both neutrophils and hepatocytes contest bacterial infection via mediating the generation of lipocalin-2 (48). In another study, miR-223, one of the most abundant miRNAs in neutrophils, has been shown to mediate neutrophil function and ameliorate alcoholic liver injury. Compared with healthy individuals, the expression of miR-223 was down-regulated in peripheral blood neutrophils from alcoholics. Mechanistic study revealed that miR-223 directly inhibited the expression of IL-6 and subsequently suppressed phagocytic oxidase p47expression with reduced ROS production in neutrophils (49).

Furthermore, the role of a matricellular proteinosteopontin (OPN) in alcohol-induced neutrophil infiltration has been intensively studied. The expressions of OPN were found to be up-regulated in the liver of mice and patients with ALD (50). Higher expression of hepatic OPN is likely to be the contributing factor of higher and early neutrophil infiltration in females, which might be the reason for increased susceptibility of females to ALD (51). Further mechanistic study showed that OPN-regulated neutrophil infiltration in the liver might result in activation of N-terminal integrin binding motif (SLAYGLR) of OPN via its receptor α_9_β_1_ (VLA9) and α_4_β_1_ (VLA4) integrins on neutrophils (52). However, in another study, it was found that OPN deficiency does not decrease the incidence of alcoholic hepatitis and expression of fibrogenic genes, but promotes the generation of IL-17a and neutrophil infiltration in mice receiving alcohol (53). This observation hints a complex role of OPN in mediating neutrophil at a different stage of ALD, deserving to be further thoroughly explored. Manipulating these mediators that participate in the pathological process of ALD via neutrophils infiltration represents the potential therapeutic approach for this disease.

Paradoxically, some studies suggested that decreased neutrophil phagocytic capacity is correlated with disease severity (54, 55). As a matter of fact, the leading cause of death in alcoholic hepatitis patients is the infection. This might be due to the severe functional failure of neutrophils mediated by endotoxemia. The overexpressed TLR2, 4, and 9 induced by bacterial products in activated neutrophil mediate the abnormalities of neutrophil function in alcoholic hepatitis (54). As the neutrophil dysfunction in alcoholic hepatitis is reversible, endotoxin-removal strategies might be new targets for ALD treatment, particularly at the advanced stage (56).

### Natural Killer Cells

Natural killer (NK) cells, a key component of innate immunity, play crucial roles in host defense against stimulus by natural cytotoxicity and secreted cytokines such as interferon-gamma (IFN-γ) production. These cells help to control the viral hepatitis, liver fibrosis, and liver tumorigenesis via directly killing infected and damaged cells, while also cause liver damage when it is over-activated. Accumulating studies have indicated that NK cells are implicated in the pathogenesis of ALD. In the chronic ethanol feeding mouse model fed with the Lieber-DeCarli liquid diet containing 5% (vol/vol) ethanol, there was a significant reduction of hepatic NK cells (57). In patients with severe alcoholic hepatitis, impaired cytotoxic functions and reduced activation of NK cells were observed (58). On the contrary, in alcoholic hepatitis patients who showed no other lesions in liver biopsy and undergoing their first episode of alcoholic hepatitis, there was a remarkable expansion of both NK cells and activated T cells in peripheral blood, associating with an increased NK cytotoxic activity (59). This discrepancy suggested that alcohol might exert different effects on NK cells at different stages of ALD. Moreover, NK cytolytic activity was constantly repressed at the stage of alcoholic cirrhosis, suggesting an off-tune immune surveillance in these patients (59). Chronic alcohol consumption accelerates liver fibrosis in patients with viral hepatitis, which might be due to the suppression of anti-fibrotic property of NK cells and interferon-gamma (IFN-γ). The inferior natural killer group 2 member D (NKG2D), TNF-related apoptosis-inducing ligand, and IFN-γ expressions on NK cells from ethanol-fed mice resulted in attenuated cytotoxicity against HSCs. Furthermore, compared with pair-fed mice, HSCs from ethanol-fed mice were resistant to NK cells killing as well as IFN-γ-induced cell cycle arrest and apoptosis. This resistance is resulting from diminished IFN-γ-activated signal transducer and activator of transcription 1 (STAT1) in HSCs, which is caused by the production of oxidative stress and the induction of suppressors of cytokine signaling proteins (60).

The mechanisms by which alcohol consumption reduces peripheral NK cell numbers and compromises cytolytic activity of NK cells have been intensively studied (61–64). It was found that chronic alcohol exposure disturbs the balance between bone marrow-derived NK cells and thymus-derived NK cells (65). The loss of splenic NK cells induced by alcohol is attributed to compromised NK cell released from the BM and enhanced splenic NK cell apoptosis (66). Chronic alcohol drinking reduced cytotoxic conventional NK cell number and its cytolytic activity by arresting these cells development at the CD27^+^CD11b^+^ stage due to the lack of IL-15 availability in the microenvironment. Supplementation with IL-15/IL-15Ralpha successfully recovered developmental defect in NK cells caused by alcohol consumption (61). A study has demonstrated that NF-κB and AP-1 partially mediate the decreased cytolytic activity of IL-2-stimulated NK cells in ethanol-treated mice through regulating transactivation of genes involved in the control of NK cells target lysis such as perforin, granzyme A, and granzyme B gene (67). Moreover, other studies has shown that alcohol consumption suppressed the cytolytic activity of NK cell partly by decreasing the function of hypothalamic β-endorphin neurons, corticotropin releasing hormone neurons, and the autonomic nervous system (68, 69). The characterization of NK cell functions would help us to better understand the pathogenesis of ALD, and indicates new therapeutic targets for managing and treating this disease.

### Natural Killer T Cells

Natural killer T (70) cells are a subset of lymphocytes that express both a T*-*(TCR) and surface receptors for NK cells, which possess characteristics of innate and adaptive immunity. There are at least two distinct subsets of NKT cells, type I and II, which recognize different lipid antigens presented by CD1d molecules. They produce cytokines associating with T helper 1 and T helper 2 cells, and also make use of Fas and TNF-α in apoptosis induction, steering the immune system into either tolerance or inflammation (71). Growing evidence showed that alcohol consumption induces peripheral and resident hepatic type I NKT cells, but not type II activation after alcohol feeding (26, 72). Type I NKT cell-induced inflammation and neutrophil recruitment lead to liver tissue damage whereas type II NKT cells show the beneficial effect on ALD via undefined mechanism (72). Chronic-plus-binge ethanol feeding increased the number of hepatic type I NKT cells and induced their activation in mice, which contributes to the development of alcoholic liver injury partially by releasing inflammatory mediators that recruit neutrophils to the liver (73). For example, IL-1β derived from Kupffer cell after alcohol exposure recruits and activates hepatic type I NKT cells, subsequently promotes neutrophil infiltration and liver inflammation, resulting in ALD (74). Another study showed that consumption of alcohol stimulates a great proportion of hepatic NKT cells and induces an elevated sensitivity of liver cells to cell-mediated lysis, resulting in severe liver injury by a mechanism that involves synchronous signals by Fas and TNF receptor-1 on hepatocytes (75). A pathway involving all-trans retinoic acid and its receptor signaling plays a vital role in suppressing activation of type I NKT cells and, consequently, attenuating alcoholic liver injury. Suppression of type I NKT cells by retinoids or activation of type II NKT cells by sulfatide counteracts the liver injury caused by alcohol (72). Although the role of NKT in ALD has been explored in growing number of animal studies, evidence from clinical research is absent. Since the CD1d pathway is well-conserved between human and mice, NKT cell subsets might be a promising target for ALD therapy and, future clinical study targeting these cells is desirable.

### Dendritic Cells

Dendritic cells (DCs) are pivotal in the coordination of innate and adaptive immune responses, activating T lymphocytes in an antigen-specific manner (76). Accumulating evidence showed that alcohol intake impairs function of DCs, which contributes to decreased adaptive immunity and increased susceptibility to pathogens (77–82). As demonstrated by both *in vitro* and *in vivo* studies, alcohol disturbs T_H_1 immune responses by inhibiting differentiation of DCs and accessory cell function via the mechanism involving reduced generation of IL-12 (83). Decreased numbers of circulating DCs and reduced secretion of inflammatory cytokines such as IL-1β, IL-6, IL-12, and TNF-α were observed in alcoholic liver cirrhosis patients (84). A significant decreased expression of an MHC class II cell surface receptor, human leukocyte antigen-DR isotype (HLADR), and an increased reactivity for CD123 were observed on peripheral blood DCs from alcoholics without significant liver injury (84). Immature DCs from alcoholics displayed fewer CD1a^+^ cells, less CD86 expression and higher HLADR expression associated with inferior endocytosis and allostimulatory functions than immature DCs from healthy individuals, indicating altered phenotype and functions of these cells. Further *in vitro* study showed that alcohol exposure prevented immature DCs from maturation, which in turn polarized naive allogeneic T cells into T_H_1 cells in response to LPS stimulation and favored a predominant T_H_2 environment (85). Co-culture of purified CD4^+^ T cells and CD11c^+^CD8α^+^ DCs derived from alcohol-fed mice exhibits reduced production of IL-6, IL-12, IL-17A, and IFN-γ and increased level of IL-13 cytokine in response to ovalbumin stimulation, indicating function alteration of this DCs subset by alcohol consumption (86). Ethanol decreased surface expression of co-stimulatory molecules (CD40, CD80, CD86) on resting or CpG-stimulated DCs subset. Ethanol-treated DCs derived from bone marrow showed inferior capacity to induce naive, allogeneic T cell proliferation and reduced ability to prime T cells *in vivo*, while liver DCs isolated from ethanol-fed mice are less affected than splenic DCs, which showed impaired functional maturation after CpG stimulation. The dysfunction of DCs induced by alcohol might be a potential mechanism by which alcohol drinking is associated with immunosuppression (87).

Mechanisms underlying the alcohol-mediated impaired ability of DCs to promote proliferation of T cell are likely associated with lack of co-stimulation and cytokine production due to diminished receptors for inflammatory mediators (88). Alcohol exposure suppresses co-stimulatory molecule CD83 expression during DCs transformation, attenuating its capacity to prime T-cell expansion (89). Monocyte-derived DCs from alcoholics showed higher levels of class I histone deacetylases (HDACs) compared to controls. HDAC inhibitors blocked alcohol-induced increased of class I HDACs and down-regulated alcohol-induced oxidative stress related genes expressed by monocyte-derived DCs (90). Additionally, the post-translational modifications in human monocyte-derived DCs after chronic alcohol exposure have been studied, and a significant increase in acetylation at H4K12 (H4K12ac) caused by alcohol was observed. Moreover, the blockage of ethanol-induced H4K12ac by inhibitor enhanced the levels of IL-15, TGF-β1, as well as TNF-α, and restored MCP-2 levels, suggesting the vital role of H4K12ac in mediating inflammation under chronic alcohol conditions (91). On the other hand, alcohol induces specifically unfolded protein response (UPR) in monocyte-derived DCs due to high amounts of ROS generated during alcohol metabolism, which may protect the DCs from oxidant injury. The expression of glycolytic enzymes, proteolytic enzymes, and chaperones hsp60 were increased to generate energy and diminish the accumulation of mis-folded proteins under the UPR. To stop mis-folded protein synthesis, the dissociation of GRP78 from stress receptors occurs. Then, an anti-apoptotic pathway by increasing the expression of anti-apoptotic proteins such as cystatin B or down-regulating pro-apoptotic factors Gal-1 and cyclophilin A might be activated in DCs to promote cell survival (92). Specific target mediating apoptosis in alcohol-induced DCs needs to be further elucidated in the future study. These targets, such as HDACs or H4K12ac, may be useful for uncovering novel therapeutic strategies for the treatment of alcohol-induced damage and may delineate different potential immune-modulatory mechanisms.

## Adaptive Immune Cells

Studies have extensively demonstrated that adaptive immunity is implicated in the pathogenesis of ALD (93). The adaptive immune system includes T cells-mediated cellular immunity and B cells-mediated humoral immunity. Specifically, CD4^+^ T helper cells are essential for the activation and differentiation of macrophages, cytotoxic CD8^+^ T cells, and B cells; CD8^+^ T cells play a critical role in eliminating cells infected with intracellular pathogens; B cells produce antibodies to remove extracellular microorganisms and prevent the spread of infections (93). Accumulating studies in animal models and humans demonstrated that alcohol abuse decreases peripheral T cells number, disrupts the balance between different T-cell types, affects activation of T cells, impedes T cells functioning, and provokes apoptosis of T cells (94). Chronic alcohol consumption also seems to induce decrease in peripheral B cells, while simultaneously promotes the generation of immunoglobulins. In particular, ALD patients show increased contents of antibodies against liver-specific autoantigens, which may promote alcohol-related hepatic injury (95). Although molecular mechanisms underlying the action of alcohol on the adaptive immune system are still incompletely elucidated, extensive studies showed that oxidative stress plays a vital role in triggering allo- and auto-immune reactions for persistent liver inflammation during the progression of ALD (96–98). Progressive ALD patients show a great prevalence of circulating immunoglobulin G (IgG) and T cells toward epitopes derived from end products of lipid peroxidation (98). The increased level of IgG against antigens derived from lipid peroxidation is associated with elevated generation of pro-inflammatory mediators, correlating with the severity of liver inflammation in both heavy drinkers and alcohol-fed animal models (98). Moreover, CYP2E1-alkylation by HER facilitates the production of anti-CYP2E1 auto-antibodies in some ALD patients (10).

### T Lymphocytes

The altered numbers of peripheral T cells resulted from alcohol abuse has been demonstrated since decades ago (99, 100). As more recent studies have identified increasing subtypes of T cells, the impact of alcohol exposure on T cells with different phenotypes has been extensively explored (101). The decreased number of peripheral blood CD4^+^/CD25^+^ T cell regulatory (102) population is associated with immune activation in alcoholic hepatitis patients, as evidenced by increased inflammatory cytokines (103). Moreover, Treg population is likely to be involved in the mechanism of hepatitis viral infection in alcoholics. Increased CD25^+^FOXP3^+^ and CD4^+^FOXP3^+^ Treg populations induced by immunization with HCV core-containing DCs from ethanol-fed mice might be responsible for the suppression of HCV core-specific CD4^+^ and CD8^+^ T-cell immune responses (104). IL-17-producing T helper 17 (T_H_17) effector cells, a subset of T helper cells distinct from T_H_1 and T_H_2 CD4^+^ T cells, were identified in the circulation and livers of patients with ALD (105). Th17 cells, which play a crucial role in controlling defensive mechanisms to bacterial infections, are increasingly recognized to promote liver neutrophil infiltration by IL-17 during alcoholic hepatitis (106). Given that a close association between T_H_17 and liver injury was observed, the T_H_17 pathway thus appears to play a vital role in ALD. Additionally, mucosa-associated invariant T cells (107), a recently identified subset of innate-like T cells, were found to be significantly depleted in ALD patients, with the consequence of increased risk of bacterial infection. It has been shown that intestinal bacterial antigens and metabolites selectively reduced MAIT cells, while did not affect CD8/CD3 T cells, indicating that the depletion and dysfunction of MAIT cells are likely due to exposure of bacteria in ALD patients (108). The hepatic expression of transcription factors that control the differentiation of MAIT cells such as RORC/RORγt, ZBTB16/PLZF, Eomes in patients with severe alcoholic hepatitis were lower than that from healthy individuals (108). The reduced number and impaired function of MAIT is associated with the bacterial infection in patients with ALD (109). Therefore, functionally reprogrammed MAIT cells *in vitro*, MAIT cells manipulation and strategies targeted at enhancing the gut barrier may signify promising immunotherapeutic approach for patients with ALD (109).

In addition to the altered number, the disrupted balance between different T cell types by alcohol exposure is intensively implicated in the development of ALD. A significant decrease in CCR5 expression on CD4^+^ T cells was found in alcoholic hepatitis, which favor imbalance of T_H_1/T_H_2 (110). Alcohol consumption reduced Treg cell population while increased T_H_17 cell number and IL-17 production. Reversing this imbalance by agents such as *Lactobacillus rhamnosus* GG supernatant could ameliorate liver injury induced by alcohol (111). Expansion of CD57^+^ T subsets in both CD4^+^ and CD8^+^ cells has been noted in ALD patients. It was found that the greater proportion of CD57^+^ T cells, the higher potential for a rapid TNF-α and IFN-γ response, showing extensive immediate T_H_1 responses in patients with ALD, rather than T_H_1 deficient as assumed. However, whether this rapid T_H_1 response has an impact on provoking cell-mediated immunity or it only promotes a rapid inflammatory response with induction of tissue damage, remain unclear (112).

Alcohol consumption also influences T-cell activation and functioning in humans and mouse models. Cytotoxic T cells present impaired cytotoxic functions and reduced activation in patients with alcoholic hepatitis, leading to severe immune incompetence (58). In alcoholic hepatitis patients whose condition seemed to improve, the frequency of IL-22-producing T helper cells was found to be increased, indicating T cell differentiation toward an IL-22-producing phenotype might be favorable for ALD suppression (113). Patients with alcoholic liver cirrhosis showed down-regulated expression of the CD28 co-stimulatory molecule, reduced ability of T cells binding to exogenous IL-2, and increased soluble CD8 levels that might interfere with CD8^+^ T cells activation. The impaired T_H_1 response and these abnormalities would affect the cytotoxic response in these patients (114). A study indicated that actively drinking patients with alcoholic cirrhosis induces T_H_1 cellular immune responses against the ethanol-oxidizing enzyme ADH, and these responses are correlated with the severity of the disease (115). The increase of ADH release from the cytoplasm of injured hepatocytes triggers autoimmune responses, forming a vicious circle to promote liver injury. These immune responses triggered by ADH may provide new targets for immune-regulatory therapies in ALD patients, and also serve as a biomarker to identify the risk of alcoholics in progressing to cirrhosis (115).

In a recent study, programmed cell death 1 (PD1), and T-cell immunoglobulin and mucin domain-containing protein 3 (TIM3), as well as their respective ligands- PD ligand 1 (PD-L1) and galectin-9, have been revealed as crucial mediators involving in innate and adaptive immunity in acute alcoholic hepatitis patients (116). T cells with an impaired function from patients with acute alcoholic hepatitis, showed higher levels of PD1 and PD-L1, or TIM3 and galectin-9, compared with that of T cells from controls (116). Antibodies against PD1 and TIM3 restored INF-γ production by T cells, decreased IL-10-producing T cells and increased antimicrobial activities of neutrophil. Blockade of PD1 and TIM3 might be the possible immune-regulation approach for ALD treatment. In our recent study, we have defined the protective role of a heterogeneous population, granulocytic-myeloid-derived suppressor cells (G-MDSCs) in response to ethanol-induced acute liver injury (3). After acute alcohol consumption, the activated T cells produce pro-inflammatory mediators and further drives the progression of alcoholic liver injury. The expansion of G-MDSCs after acute alcohol administration alleviated alcohol-caused hepatic injury via suppressing T cells activation. The role of G-MDSCs in the chronic setting of ALD should be thoroughly elucidated, which might be a novel potential target for immune-regulatory treatment of ALD in the future.

### B Lymphocytes

B cells, a subtype of lymphocytes derived from the bone marrow, produce immunoglobulins located either on the cell surface or secreted as antibodies, which mediates both T cells-dependent and -independent immune responses (117). The B cells number was lower in heavy alcoholics than that of moderate or light drinkers. In alcoholics with ALD, the loss of circulating B cells is particularly severe (101, 118). Moreover, alcohol exposure can affect the differentiation of progenitor B cells at a late stage by down-regulating the expression level of related transcription factors and cytokine receptors (119). Increased circulating level of immunoglobulins is a characteristic of alcoholic liver cirrhosis patients, which might be attributed to TLR-9 priming of B cells (120). TLR pathways are involved in the maturation and immunoglobulins synthesis of B cells. However, another study showed that short-term ethanol treatment at high dose down-regulated splenic macrophages and DCs activity via enhancing B cells function as the antigen-presenting cell, and eventually facilitating a microenvironment that leads to increased activation of CD4^+^ T cells (121). The roles of B cells within the spectrum of ALD deserve to be further elucidated with mechanistic studies required.

## Potential Therapeutic Approaches to Treat ALD via Regulation of Immune Response

**Figure 2:**
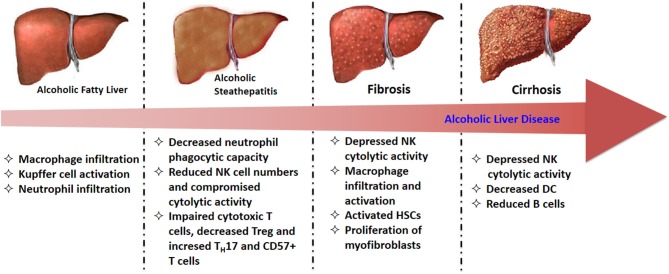
The alterations of immune cells in diverse stages of ALD.

The alterations of immune cells in the progressive stage of ALD are summarized in Figure 2. In the early stage of ALD, the activated Kupffer cells induced by increased hepatic LPS produce a large amount of ROS, as well as pro-inflammatory cytokines and chemokines, which finally result in liver injury (122). Then those chemokine and cytokines such as MCP-1, macrophage inflammatory protein-(MIP)-1α, and MIP-1β increased macrophage and neutrophil numbers in fatty liver and steatosis (21). In alcoholic hepatitis, although neutrophils are primed and infiltrate the liver to induce damage, their phagocytic capacities are significantly reduced (56). Moreover, NK cell numbers as well as their cytolytic activity are also decreased in alcoholic hepatitis. The compromised cytolytic activity is due to the reduced expression of tumor necrosis factor-related apoptosis-inducing ligand (TRAIL), natural killer group 2D (NKG2D), and IFN-γ on NK cells after chronic alcohol exposure, subsequently abrogated the killing effects of NK cells on activated HSCs (123). The expression of NKG2D on cytotoxic T cells from alcoholic hepatitis patients was also observed to be decreased, resulting in impaired cytotoxic function. These comprised immune functions lead to a high infection rate in patients with alcoholic hepatitis. In alcoholic fibrosis, one of the most remarkable feature is the activated HSCs and proliferation of myofibroblasts stimulated by LPS and TGF-β1 (124). Increasing evidence has indicated that the activation of NK cells could inhibit the pro-fibrotic progress via killing HSCs (125). In alcoholic cirrhosis, both amplified systemic inflammation and immunodeficiency were observed. The immunodeficiency is mainly attributed to the depressed NK cytolytic activity, decreased DC and reduced B cells. Generally, the extent of immune dysfunction is closely related to severity of liver injury, and the maximum immunodeficiency is observed in patients with alcoholic cirrhosis (11).

Given the vital role of immune cells in the pathogenesis process of ALD, interventions targeting the immune response have been intensively studied. We have summarized the emerging mediators that are involved in the response of several immune cells in ALD from studies in recent several years (Table 1). These mediators could be targeted to prevent or alleviate alcoholic liver injury. Adiponectin, an adipokine mainly secreted by adipocytes, has been shown to inhibit alcohol-induced ROS production via regulating NADPH oxidase in macrophages, playing a protective role against chronic alcohol exposure (126). Globular adiponectin inhibited activation of NADPH oxidase and increased expression of NADPH oxidase subunits including Nox2 and p22 by mediating NF-κB pathway. Further mechanistic study revealed that globular adiponectin-induced the phosphorylation of liver kinase B1 (LKB1), which is an upstream signaling molecule mediating AMPK activation, to suppress the activation of NADPH oxidase induced by alcohol in macrophages (126). Additionally, the impact of ethanol on the adipose secretome has been demonstrated to be closely associated with the cargo of adipocyte-derived extracellular vesicles and anaphylatoxins complement component 5a receptor-1 (C5aR1) was involved in this process (128). IL-1 receptor like 1 (ST2) showed a beneficial role in preventing alcoholic liver injury at early and middle stages of ALD. It restrains the inflammatory activation of Kupffer cells via suppressing the NF-κB pathway. In the case of severe alcoholic liver injury with significant cell death, however, the release of IL-33 from dead cells that acts through canonical ST2 signaling in hepatic macrophage exacerbated hepatic injury (127). Retinoic acid, is found to be depleted in ALD, with the consequence of priming pro-inflammatory cytokines production. The supplementation of retinoic acid in alcohol-treated animals reduced the stability of TNF-α mRNA in hepatic macrophage, thus alleviating liver injury (129). Beta-hydroxybutyrate (BHB), one of the main ketone body, was found to show anti-inflammatory and hepatoprotective effects via an Hcar2 dependent pathway. In patients with alcoholic hepatitis, the loss of BHB occurs. Supplementation of BHB displayed significant therapeutic effect on preventing alcoholic liver injury (130). BHB treatment increased the M2 phenotype of intrahepatic macrophage and promoted hepatic IL-10 transcripts. *In vitro* study also indicated that BHB induced lower mitochondrial membrane potential, resulting in improved transcriptional level of M2 related genes in bone marrow derived macrophages (130). This finding supports the concept of metabolite-based therapy for alcoholic hepatitis.

**Table 1:** Emerging mediators involved in the response of several immune cells in ALD.

**Mediators/Targets**	**Involved cells**	**Effects**	**Pathways**	**References**
Kruppel-like factor 4 (KLF4)	Macrophage	Regulate M1/M2 macrophage polarization	Activation of NF-κB	(25)
Macrophage migration inhibitory factor (26)	F4/80+ macrophages	Sensitize mice to LPS and promote alcoholic liver injury	Recruit infiltrating monocytes and inflammatory cytokine production	(29)
Extracellular vesicles	Kupffer cell	Increase TNF-α^+^ IL-12/23^+^ M1 Kupffer cells and decrease CD206^+^CD163^+^ M2 Kupffer cells, lead to inflammation activation	Hsp90 and NF-κB pathway	(33)
Telomerase reverse transcriptase (23)	Macrophage	Switch macrophages toward M1 phenotype	Activation of NF-κB pathway	(24)
miR-155	Macrophage	Promote macrophage activation and facilitate alcohol-induced injury and	Production of TNF-α via enhancing mRNA stability	(31)
Cannabinoid CB2 receptors	Kupffer cells	Regulate M1/M2 balance in Kupffer cells, protect liver from alcoholic injury	Via an autophagy-dependent manner via by HO-1 induction	(37, 38)
Adiponectin	Macrophage	Inhibit alcohol-induced ROS production, protective role against chronic alcohol exposure	Inhibit activation of NADPH oxidase and the increased expression of NADPH oxidase subunits including Nox2 and p22 by mediating NF-κB pathway, induce the phosphorylation of LKB1	(126)
IL-1 receptor like 1 (ST2)	Kupffer cells	Prevent alcoholic liver injury at early and mild stages of ALD, restrain the inflammatory activation of Kupffer cells	Suppress NF-κB pathway	(127)
Human neutrophil peptide (HNP)-1	Neutrophils	Exacerbate alcohol-induced liver fibrosis and hepatocyte apoptosis	Via down-regulating Bcl2 expression and up-regulating miR-34a-5p expression	(41)
Intercellular adhesion molecule 1 (ICAM-1)	Neutrophils	Exacerbate alcoholic liver injury	Promote neutrophil infiltration	(42)
E-selectin	Neutrophils	Exacerbate alcoholic liver injury, activation of neutrophils	Promote transition from slow rolling to arrest, increase the efficient transendothelial migration	(43)
CCL2	Neutrophils	Significantly increased in alcoholic hepatitis patients	IL-8, neutrophil recruitment	(45)
Albumin	Neutrophils	Induce hepatic oxidative stress and inflammation	Neutrophils activation	(44)
CXCL1	Neutrophils; hepatocytes; HSCs	Neutrophil infiltration, lead to alcoholic liver injury	TLR2 and TLR9-dependent MyD88-dependent pathway, CXCR2	(46)
Lipocalin-2	Neutrophils	Propagate the development of ALD	Drive neutrophil infiltration	(47)
miR-233	Neutrophils	Mediate neutrophil function and ameliorate alcoholic liver injury	Inhibite IL-6 and subsequently suppressed phagocytic oxidase p47 expression with reduced ROS production in neutrophils	(49)
Osteopontin	Neutrophils	Regulate neutrophil infiltration	Signaling by N-terminal integrin binding motif (SLAYGLR) of OPN via its receptor α_9_β_1_ (VLA9) and α_4_β_1_ (VLA4) integrins on neutrophils	(52, 53)
IL-15/IL-15R alpha	NK cells	Increase cytotoxic conventional NK cell number and cytolytic activity	Inhibit the arrest of NK cell development at the CD27^+^CD11b^+^ stage due to a lack of IL-15 availability in the microenvironment	(61)
IL-1β	Type I NKT cells	Lead to liver inflammation	Promote neutrophil infiltration	(74)
All-trans retinoic acid	Type I NKT cells	Attenuate alcoholic liver injury	Suppressing activation of type I NKT cells	(72)
Class I histone deacetylases (HDACs)	DC	Promote alcoholic liver injury	Regulate alcohol-induced oxidative stress related genes expressed by monocyte-derived DC	(90)
H4K12ac	Human monocyte-derived DC	Mediate inflammation	Decrease the levels of IL-15, TGF-β1, TNF-α, and regulate MCP-2 levels	(91)
IL-17	T_H_17	Exacerbate alcoholic liver injury	Promote liver neutrophil infiltration	(106)
IL-22	T helper cells	Attenuate alcoholic liver injury	Inhibit inflammation	(113)
PD1 and TIM3	T cells	Drive alcoholic liver injury	Involve in INF-γ production by T cells, promote IL-10-producing T cells, and affect antimicrobial activities of neutrophil	(116)

Several clinic trials of drugs that target those mediators for alcoholic patients are on-going or has been performed (refer to http://www.ClinicalTrials.gov). For example, anakinra, IL-1 receptor antagonist, has been tested in patients with severe acute alcoholic hepatitis in a double-blind randomized controlled trial (NCT01809132). It showed the potential to decrease the associated inflammation, to enhance gut barrier function, and to inhibit the progress of hepatorenal syndrome. Clinic trials of granulocyte-colony stimulating factors (G-CSF) on alcoholic hepatitis have been intensively performed in recent years. Two studies (NCT01341951/NCT01820208) have indicated the survival benefits with the use of G-CSF in patients with alcoholic hepatitis. In several on-going clinical studies, granulocyte-colony stimulating factors (G-CSF) is used in alcoholic hepatitis patients to examine its safety and efficacy (NCT03703674/ NCT02442180). The safety and efficacy of combination therapy of G-CSF and N-acetyl cysteine are also tested in the patients with alcoholic hepatitis by a randomized clinic trial (NCT02971306). In the future, more clinic trials testing agents which mediate immunity in patients with ALD are expected.

Since therapeutic approaches for ALD are still limited and unsatisfactory, Chinese herbal medicine has been considered as a promising alternative approach (131–135). Chinese herbal medicine used in treating liver injury is principally based on eliminating toxins, increasing blood circulation, resolving stasis, and improving host immunity (136–138). Several herbal medicines have been demonstrated to prevent alcoholic liver injury via immunoregulation by modern research. For example, silymarin has been found to significantly promote the survival period of alcoholic cirrhosis patients (139, 140). Further mechanism study indicated that flavonoids from *Silybum marianum*, including silybin and silymarin, could normalize immunoregulatory defects *via* restorating the cellular thiol status, inducing T-cell activation (CD69) with a significant reduction of TNF (139, 141). Although there is still a long way to go to fulfill the immunity treatment in ALD, it represents a very encouraging field for ALD therapy.

## Conclusions and Perspectives

In conclusion, accumulating studies showed a complex interaction between alcohol and the immune systems, which might increase patient's susceptibility to damage. On one hand, at the early stage of ALD, activation of innate immunity induced by alcohol in liver precipitates disorders ranging from localized and temporary inflammation to extensive hepatocellular damage and liver injury. Adaptive immune response triggered by oxidative modification of hepatic constituents conversely favors the stimulation of innate immune response, contributing to the sustained hepatic inflammation in ALD. On the other hand, the suppressed antigen-presenting capability of various innate immune cell types, impaired proliferation, and function of T cells indicates the off-tune immune system that may consequently lead to increased risk of infection, especially in patients with advanced ALD. The failure in the liver's function to remove microbial and other circulating pro-inflammatory metabolites, as well as release of immunogenic cellular debris from necrotic hepatocytes, consequently cause persistent activation of immune pathways, which aggravates state of the illness. To define the precise pathogenic role of these immune cells in the context of ALD, more prospective clinical studies that further elucidate their phenotypic diversity are expected. Future studies should focus on understanding how alcohol affects the population, phenotypic switch, and functions of hepatic immune cells. The interaction between diverse immune cells within ALD spectrum, and if targeting those cells relieves hepatic injury, will deliver promising strategies to manage patients with ALD (21). Moreover, identification of specific immune component at different stages of ALD might facilitate the discrimination of alcoholics in risk of progression to alcoholic hepatitis, fibrosis or cirrhosis.

## Author Contributions

SL wrote the manuscript. NW and H-YT revised the manuscript. XW and YigF commented on the manuscript and discussed the manuscript. YibF designed, revised and finalized the manuscript.

### Conflict of Interest Statement

The authors declare that the research was conducted in the absence of any commercial or financial relationships that could be construed as a potential conflict of interest.

## References

[1] Li W Amet T Xing Y Yang D Liangpunsakul S Puri P. Alcohol abstinence ameliorates the dysregulated immune profiles in patients with alcoholic hepatitis: a prospective observational study. Hepatology. (2017) 66:575–90. 10.1002/hep.29242 28466561 PMC5548491

[2] Byun JS Yi HS. Hepatic immune microenvironment in alcoholic and nonalcoholic liver disease. Biomed Res Int. (2017) 2017:6862439. 10.1155/2017/6862439 28852648 PMC5567444

[3] Li S Wang N Tan HY Hong M Yuen MF Li H. Expansion of granulocytic, myeloid-derived suppressor cells in response to ethanol-induced acute liver damage. Front Immunol. (2018) 9:1524. 10.3389/fimmu.2018.01524 30072984 PMC6060237

[4] Petrasek J Iracheta-Vellve A Saha B Satishchandran A Kodys K Fitzgerald KA. Metabolic danger signals, uric acid and ATP, mediate inflammatory cross-talk between hepatocytes and immune cells in alcoholic liver disease. J Leukoc Biol. (2015) 98:249–56. 10.1189/jlb.3AB1214-590R 25934928 PMC4501673

[5] Dhanda AD Collins PL. Immune dysfunction in acute alcoholic hepatitis. World J Gastroenterol. (2015) 21:11904–13. 10.3748/wjg.v21.i42.11904 26576079 PMC4641112

[6] Mandrekar P Ambade A. Immunity and inflammatory signaling in alcoholic liver disease. Hepatol Int. (2014) 8(Suppl. 2):439–46. 10.1007/s12072-014-9518-8 26201323 PMC4587491

[7] Szabo G Petrasek J Bala S. Innate immunity and alcoholic liver disease. Dig Dis. (2012) 30(Suppl. 1):55–60. 10.1159/000341126 23075869 PMC6412139

[8] Albano E Vidali M. Immune mechanisms in alcoholic liver disease. Genes Nutr. (2010) 5:141–7. 10.1007/s12263-009-0151-4 19809845 PMC2885168

[9] Muhanna N Doron S Wald O Horani A Eid A Pappo O. Activation of hepatic stellate cells after phagocytosis of lymphocytes: a novel pathway of fibrogenesis. Hepatology. (2008) 48:963–77. 10.1002/hep.22413 18726940 PMC2880478

[10] Barnes MA Roychowdhury S Nagy LE. Innate immunity and cell death in alcoholic liver disease: role of cytochrome P4502E1. Redox Biol. (2014) 2:929–35. 10.1016/j.redox.2014.07.007 25180169 PMC4143810

[11] Duddempudi AT. Immunology in alcoholic liver disease. Clin Liver Dis. (2012) 16:687–98. 10.1016/j.cld.2012.08.003 23101977

[12] Nagy LE. The role of innate immunity in alcoholic liver disease. Alcohol Res. (2015) 37:237–50. 26695748 10.35946/arcr.v37.2.08 PMC4590620

[13] Suh YG Jeong WI. Hepatic stellate cells and innate immunity in alcoholic liver disease. World J Gastroenterol. (2011) 17:2543–51. 10.3748/wjg.v17.i20.2543 21633659 PMC3103812

[14] Szabo G Mandrekar P Petrasek J Catalano D. The unfolding web of innate immune dysregulation in alcoholic liver injury. Alcohol Clin Exp Res. (2011) 35:782–6. 10.1111/j.1530-0277.2010.01398.x 21284666 PMC3742381

[15] Thakur V McMullen MR Pritchard MT Nagy LE. Regulation of macrophage activation in alcoholic liver disease. J Gastroenterol Hepatol. (2007) 22(Suppl. 1):S53–6. 10.1111/j.1440-1746.2006.04650.x 17567466

[16] Thurman RG. Alcoholic liver injury involves activation of Kupffer cells by endotoxin. Am J Physiol. (1998) 275(4 Pt 1):G605–11. 10.1152/ajpgi.1998.275.4.G605 9756487

[17] Wheeler MD Kono H Yin M Nakagami M Uesugi T Arteel GE. The role of Kupffer cell oxidant production in early ethanol-induced liver disease. Free Radic Biol Med. (2001) 31:1544–9. 10.1016/S0891-5849(01)00748-1 11744328

[18] Sandahl TD Grønbaek H Møller HJ Støy S Thomsen KL Dige AK. Hepatic macrophage activation and the LPS pathway in patients with alcoholic hepatitis: a prospective cohort study. Am J Gastroenterol. (2014) 109:1749–56. 10.1038/ajg.2014.262 25155228

[19] Wang M You Q Lor K Chen F Gao B Ju C. Chronic alcohol ingestion modulates hepatic macrophage populations and functions in mice. J Leukoc Biol. (2014) 96:657–65. 10.1189/jlb.6A0114-004RR 25030420 PMC4163632

[20] Lavin Y Mortha A Rahman A Merad M. Regulation of macrophage development and function in peripheral tissues. Nat Rev Immunol. (2015) 15:731–44. 10.1038/nri3920 26603899 PMC4706379

[21] Ju C Mandrekar P. Macrophages and Alcohol-Related Liver Inflammation. Alcohol Res. (2015) 37:251–62. 26717583 10.35946/arcr.v37.2.09 PMC4590621

[22] Lee J French B Morgan T French SW. The liver is populated by a broad spectrum of markers for macrophages. In alcoholic hepatitis the macrophages are M1 and M2. Exp Mol Pathol. (2014) 96:118–25. 10.1016/j.yexmp.2013.09.004 24145004 PMC3944995

[23] Bracken K Askie L Keech AC Hague W Wittert G. Recruitment strategies in randomised controlled trials of men aged 50 years and older: a systematic review. BMJ Open. (2019) 9:e025580. 10.1136/bmjopen-2018-025580 30948584 PMC6500287

[24] Wu XQ Yang Y Li WX Cheng YH Li XF Huang C. Telomerase reverse transcriptase acts in a feedback loop with NF-kappaB pathway to regulate macrophage polarization in alcoholic liver disease. Sci Rep. (2016) 6:18685. 10.1038/srep18685 26725521 PMC4698632

[25] Saha B Bala S Hosseini N Kodys K Szabo G. Kruppel-like factor 4 is a transcriptional regulator of M1/M2 macrophage polarization in alcoholic liver disease. J Leukoc Biol. (2015) 97:963–73. 10.1189/jlb.4A1014-485R 25740962 PMC6608000

[26] Chelakkot-Govindalayathil AL Mifuji-Moroka R D'Alessandro-Gabazza CN Toda M Matsuda Y Gil-Bernabe P. Protein S exacerbates alcoholic hepatitis by stimulating liver natural killer T cells. J Thromb Haemost. (2015) 13:142–54. 10.1111/jth.12789 25399514

[27] Marin V Poulsen K Odena G McMullen MR Altamirano J Sancho-Bru P. Hepatocyte-derived macrophage migration inhibitory factor mediates alcohol-induced liver injury in mice and patients. J Hepatol. (2017) 67:1018–25. 10.1016/j.jhep.2017.06.014 28647568 PMC5650516

[28] Kumagi T Akbar F Horiike N Onji M. Increased serum levels of macrophage migration inhibitory factor in alcoholic liver diseases and their expression in liver tissues. Clin Biochem. (2001) 34:189–93. 10.1016/S0009-9120(01)00214-4 11408016

[29] Barnes MA McMullen MR Roychowdhury S Pisano SG Liu X Stavitsky AB. Macrophage migration inhibitory factor contributes to ethanol-induced liver injury by mediating cell injury, steatohepatitis, and steatosis. Hepatology. (2013) 57:1980–91. 10.1002/hep.26169 23174952 PMC3597752

[30] Xiong S She H Zhang AS Wang J Mkrtchyan H Dynnyk A. Hepatic macrophage iron aggravates experimental alcoholic steatohepatitis. Am J Physiol Gastrointest Liver Physiol. (2008) 295:G512–21. 10.1152/ajpgi.90327.2008 18599584 PMC2536779

[31] Bala S Marcos M Kodys K Csak T Catalano D Mandrekar P. Up-regulation of microRNA-155 in macrophages contributes to increased tumor necrosis factor {alpha} (TNF{alpha}) production via increased mRNA half-life in alcoholic liver disease. J Biol Chem. (2011) 286:1436–44. 10.1074/jbc.M110.145870 21062749 PMC3020752

[32] Gobejishvili L Barve S Joshi-Barve S Uriarte S Song Z McClain C. Chronic ethanol-mediated decrease in cAMP primes macrophages to enhanced LPS-inducible NF-kappaB activity and TNF expression: relevance to alcoholic liver disease. Am J Physiol Gastrointest Liver Physiol. (2006) 291:G681–8. 10.1152/ajpgi.00098.2006 16751174

[33] Saha B Momen-Heravi F Furi I Kodys K Catalano D Gangopadhyay A. Extracellular vesicles from mice with alcoholic liver disease carry a distinct protein cargo and induce macrophage activation through heat shock protein 90. Hepatology. (2018) 67:1986–2000. 10.1002/hep.29732 29251792 PMC5906190

[34] Ambade A Catalano D Lim A Kopoyan A Shaffer SA Mandrekar P. Inhibition of heat shock protein 90 alleviates steatosis and macrophage activation in murine alcoholic liver injury. J Hepatol. (2014) 61:903–11. 10.1016/j.jhep.2014.05.024 24859453 PMC4169725

[35] Horiguchi N Wang L Mukhopadhyay P Park O Jeong WI Lafdil F. Cell type-dependent pro- and anti-inflammatory role of signal transducer and activator of transcription 3 in alcoholic liver injury. Gastroenterology. (2008) 134:1148–58. 10.1053/j.gastro.2008.01.016 18395093 PMC2376046

[36] Mandal P Pritchard MT Nagy LE. Anti-inflammatory pathways and alcoholic liver disease: role of an adiponectin/interleukin-10/heme oxygenase-1 pathway. World J Gastroenterol. (2010) 16:1330–6. 10.3748/wjg.v16.i11.1330 20238399 PMC2842524

[37] Louvet A Teixeira-Clerc F Chobert MN Deveaux V Pavoine C Zimmer A. Cannabinoid CB2 receptors protect against alcoholic liver disease by regulating Kupffer cell polarization in mice. Hepatology. (2011) 54:1217–26. 10.1002/hep.24524 21735467

[38] Denaës T Lodder J Chobert MN Ruiz I Pawlotsky JM Lotersztajn S. The Cannabinoid receptor 2 protects against alcoholic liver disease via a macrophage autophagy-dependent pathway. Sci Rep. (2016) 6:28806. 10.1038/srep28806 27346657 PMC4921859

[39] Taïeb J Delarche C Paradis V Mathurin P Grenier A Crestani B. Polymorphonuclear neutrophils are a source of hepatocyte growth factor in patients with severe alcoholic hepatitis. J Hepatol. (2002) 36:342–8. 10.1016/S0168-8278(01)00276-8 11867177

[40] Jaeschke H. Neutrophil-mediated tissue injury in alcoholic hepatitis. Alcohol. (2002) 27:23–7. 10.1016/S0741-8329(02)00200-8 12062633

[41] Ibusuki R Uto H Oda K Ohshige A Tabu K Mawatari S. Human neutrophil peptide-1 promotes alcohol-induced hepatic fibrosis and hepatocyte apoptosis. PLoS ONE. (2017) 12:e0174913. 10.1371/journal.pone.0174913 28403148 PMC5389644

[42] Woodfin A Voisin MB Nourshargh S. Recent developments and complexities in neutrophil transmigration. Curr Opin Hematol. (2010) 17:9–17. 10.1097/MOH.0b013e3283333930 19864945 PMC2882030

[43] Bertola A Park O Gao B. Chronic plus binge ethanol feeding synergistically induces neutrophil infiltration and liver injury in mice: a critical role for E-selectin. Hepatology. (2013) 58:1814–23. 10.1002/hep.26419 23532958 PMC3726575

[44] Das S Maras JS Hussain MS Sharma S David P Sukriti S. Hyperoxidized albumin modulates neutrophils to induce oxidative stress and inflammation in severe alcoholic hepatitis. Hepatology. (2017) 65:631–46. 10.1002/hep.28897 27775820

[45] Degré D Lemmers A Gustot T Ouziel R Trépo E Demetter P. Hepatic expression of CCL2 in alcoholic liver disease is associated with disease severity and neutrophil infiltrates. Clin Exp Immunol. (2012) 169:302–10. 10.1111/j.1365-2249.2012.04609.x 22861370 PMC3445007

[46] Roh YS Zhang B Loomba R Seki E. TLR2 and TLR9 contribute to alcohol-mediated liver injury through induction of CXCL1 and neutrophil infiltration. Am J Physiol Gastrointest Liver Physiol. (2015) 309:G30–41. 10.1152/ajpgi.00031.2015 25930080 PMC4491507

[47] Wieser V Tymoszuk P Adolph TE Grander C Grabherr F Enrich B. Lipocalin 2 drives neutrophilic inflammation in alcoholic liver disease. J Hepatol. (2016) 64:872–80. 10.1016/j.jhep.2015.11.037 26682726

[48] Li H Feng D Cai Y Liu Y Xu M Xiang X. Hepatocytes and neutrophils cooperatively suppress bacterial infection by differentially regulating lipocalin-2 and neutrophil extracellular traps. Hepatology. (2018) 68:1604–20. 10.1002/hep.29919 29633303 PMC6173649

[49] Li M He Y Zhou Z Ramirez T Gao Y Gao Y MicroRNA-223 ameliorates alcoholic liver injury by inhibiting the IL-6-p47(phox)-oxidative stress pathway in neutrophils. Gut. (2017) 66:705–15. 10.1136/gutjnl-2016-311861 27679493 PMC5458746

[50] Apte UM Banerjee A McRee R Wellberg E Ramaiah SK. Role of osteopontin in hepatic neutrophil infiltration during alcoholic steatohepatitis. Toxicol Appl Pharmacol. (2005) 207:25–38. 10.1016/j.taap.2004.12.018 15885730

[51] Banerjee A Apte UM Smith R Ramaiah SK. Higher neutrophil infiltration mediated by osteopontin is a likely contributing factor to the increased susceptibility of females to alcoholic liver disease. J Pathol. (2006) 208:473–85. 10.1002/path.1917 16440289

[52] Banerjee A Lee JH Ramaiah SK Interaction of osteopontin with neutrophil α_4_β_1_ and α_9_β_1_ integrins in a rodent model of alcoholic liver disease. Toxicol Appl Pharmacol. (2008) 233:238–46. 10.1016/j.taap.2008.08.008 18778724

[53] Lazero R Wu R Lee S Zhu NL Chen CL French SW Osteopontin deficiency does not prevent but promotes alcoholic neutrophilic hepatitis in mice. Hepatology. (2015) 61:129–40. 10.1002/hep.27383 25132354 PMC4280361

[54] Stadlbauer V Mookerjee RP Wright GA Davies NA Jürgens G Hallström S. Role of Toll-like receptors 2, 4, and 9 in mediating neutrophil dysfunction in alcoholic hepatitis. Am J Physiol Gastrointest Liver Physiol. (2009) 296:G15–22. 10.1152/ajpgi.90512.2008 19033535 PMC2636930

[55] Rajkovic IA Williams R. Mechanisms of abnormalities in host defences against bacterial infection in liver disease. Clin Sci (Lond). (1985) 68:247–53. 10.1042/cs0680247 2982530

[56] Mookerjee RP Stadlbauer V Lidder S Wright GA Hodges SJ Davies NA. Neutrophil dysfunction in alcoholic hepatitis superimposed on cirrhosis is reversible and predicts the outcome. Hepatology. (2007) 46:831–40. 10.1002/hep.21737 17680644

[57] Pan HN Sun R Jaruga B Hong F Kim WH Gao B. Chronic ethanol consumption inhibits hepatic natural killer cell activity and accelerates murine cytomegalovirus-induced hepatitis. Alcohol Clin Exp Res. (2006) 30:1615–23. 10.1111/j.1530-0277.2006.00194.x 16930225

[58] Støy S Dige A Sandahl TD Laursen TL Buus C Hokland M. Cytotoxic T lymphocytes and natural killer cells display impaired cytotoxic functions and reduced activation in patients with alcoholic hepatitis. Am J Physiol Gastrointest Liver Physiol. (2015) 308:G269–76. 10.1152/ajpgi.00200.2014 25501547

[59] Laso FJ Madruga JI López A Ciudad J Alvarez-Mon M San Miguel J. Abnormalities of peripheral blood T lymphocytes and natural killer cells in alcoholic hepatitis persist after a 3-month withdrawal period. Alcohol Clin Exp Res. (1997) 21:672–6. 10.1111/j.1530-0277.1997.tb03821.x 9194923

[60] Jeong WI Park O Gao B. Abrogation of the antifibrotic effects of natural killer cells/interferon-gamma contributes to alcohol acceleration of liver fibrosis. Gastroenterology. (2008) 134:248–58. 10.1053/j.gastro.2007.09.034 18166357 PMC2923436

[61] Zhang F Little A Zhang H Chronic alcohol consumption inhibits peripheral NK cell development and maturation by decreasing the availability of IL-*J Leukoc Biol*. (2017) 101:1015–27. 10.1189/jlb.1A0716-298RR PMC5346179 27837016

[62] Meadows GG Blank SE Duncan DD. Influence of ethanol consumption on natural killer cell activity in mice. Alcohol Clin Exp Res. (1989) 13:476–9. 10.1111/j.1530-0277.1989.tb00359.x 2679200

[63] Zhang T Guo CJ Douglas SD Metzger DS O'Brien CP Li Y. Alcohol suppresses IL-2-induced CC chemokine production by natural killer cells. Alcohol Clin Exp Res. (2005) 29:1559–67. 10.1097/01.alc.0000179364.32003.9f 16205356 PMC4015110

[64] Ben-Eliyahu S Page GG Yirmiya R Taylor AN. Acute alcohol intoxication suppresses natural killer cell activity and promotes tumor metastasis. Nat Med. (1996) 2:457–60. 10.1038/nm0496-457 8597957

[65] Zhang H Meadows GG. Chronic alcohol consumption perturbs the balance between thymus-derived and bone marrow-derived natural killer cells in the spleen. J Leukoc Biol. (2008) 83:41–7. 10.1189/jlb.0707472 17906116

[66] Zhang H Meadows GG. Exogenous IL-15 in combination with IL-15R alpha rescues natural killer cells from apoptosis induced by chronic alcohol consumption. Alcohol Clin Exp Res. (2009) 33:419–27. 10.1111/j.1530-0277.2008.00852.x 19120059 PMC2651996

[67] Zhou J Meadows GG. Alcohol consumption decreases IL-2-induced NF-kappaB activity in enriched NK cells from C57BL/6 mice. Toxicol Sci. (2003) 73:72–9. 10.1093/toxsci/kfg047 12700414

[68] Boyadjieva N Advis JP Sarkar DK. Role of beta-endorphin, corticotropin-releasing hormone, and autonomic nervous system in mediation of the effect of chronic ethanol on natural killer cell cytolytic activity. Alcohol Clin Exp Res. (2006) 30:1761–7. 10.1111/j.1530-0277.2006.00209.x 17010143

[69] Dokur M Boyadjieva NI Advis JP Sarkar DK. Modulation of hypothalamic beta-endorphin-regulated expression of natural killer cell cytolytic activity regulatory factors by ethanol in male Fischer-344 rats. Alcohol Clin Exp Res. (2004) 28:1180–6. 10.1097/01.ALC.0000134222.20309.71 15318116

[70] Balato A Unutmaz D Gaspari AA. Natural killer T cells: An unconventional T-cell subset with diverse effector and regulatory functions. J Invest Dermatol. (2009). 129:1628–42. 10.1038/jid.2009.30 19262602

[71] Kremer M Hines IN. Natural killer T cells and non-alcoholic fatty liver disease: fat chews on the immune system. World J Gastroenterol. (2008) 14:487–8. 10.3748/wjg.14.487 18200676 PMC2679142

[72] Maricic I Sheng H Marrero I Seki E Kisseleva T Chaturvedi S. Inhibition of type I natural killer T cells by retinoids or following sulfatide-mediated activation of type II natural killer T cells attenuates alcoholic liver disease in mice. Hepatology. (2015) 61:1357–69. 10.1002/hep.27632 25477000 PMC4376634

[73] Mathews S Feng D Maricic I Ju C Kumar V Gao B. Invariant natural killer T cells contribute to chronic-plus-binge ethanol-mediated liver injury by promoting hepatic neutrophil infiltration. Cell Mol Immunol. (2016) 13:206–16. 10.1038/cmi.2015.06 25661730 PMC4786627

[74] Cui K Yan G Xu C Chen Y Wang J Zhou R. Invariant NKT cells promote alcohol-induced steatohepatitis through interleukin-1beta in mice. J Hepatol. (2015) 62:1311–8. 10.1016/j.jhep.2014.12.027 25582105

[75] Minagawa M Deng Q Liu ZX Tsukamoto H Dennert G. Activated natural killer T cells induce liver injury by Fas and tumor necrosis factor-alpha during alcohol consumption. Gastroenterology. (2004) 126:1387–99. 10.1053/j.gastro.2004.01.022 15131799

[76] Aloman C Friedman SL Merad M. Dendritic cells in alcoholic liver injury and fibrosis. Alcohol Clin Exp Res. (2011) 35:776–81. 10.1111/j.1530-0277.2010.01397.x 21284665

[77] Szabo G Catalano D White B Mandrekar P. Acute alcohol consumption inhibits accessory cell function of monocytes and dendritic cells. Alcohol Clin Exp Res. (2004) 28:824–8. 10.1097/01.ALC.0000127104.80398.9B 15166660

[78] Lau AH Thomson AW Colvin BL. Chronic ethanol exposure affects *in vivo* migration of hepatic dendritic cells to secondary lymphoid tissue. Hum Immunol. (2007) 68:577–85. 10.1016/j.humimm.2007.03.008 17584579

[79] Edsen-Moore MR Fan J Ness KJ Marietta JR Cook RT Schlueter AJ. Effects of chronic ethanol feeding on murine dendritic cell numbers, turnover rate, and dendropoiesis. Alcohol Clin Exp Res. (2008) 32:1309–20. 10.1111/j.1530-0277.2008.00699.x 18540909 PMC2553395

[80] Feng D Eken A Ortiz V Wands JR. Chronic alcohol-induced liver disease inhibits dendritic cell function. Liver Int. (2011) 31:950–63. 10.1111/j.1478-3231.2011.02514.x 21733084 PMC3967860

[81] Zwolak A Jastrzebska I Surdacka A Kasztelan-Szczerbinska B Łozowski CT Rolinski J. Peripheral blood dendritic cells in alcoholic and autoimmune liver disorders. Hum Exp Toxicol. (2012) 31:438–46. 10.1177/0960327111426582 22076495

[82] Rendon JL Janda BA Bianco ME Choudhry MA. Ethanol exposure suppresses bone marrow-derived dendritic cell inflammatory responses independent of TLR4 expression. J Interferon Cytokine Res. (2012) 32:416–25. 10.1089/jir.2012.0005 22812678 PMC3438840

[83] Mandrekar P Catalano D Dolganiuc A Kodys K Szabo G. Inhibition of myeloid dendritic cell accessory cell function and induction of T cell anergy by alcohol correlates with decreased IL-12 production. J Immunol. (2004) 173:3398–407. 10.4049/jimmunol.173.5.3398 15322204

[84] Laso FJ Vaquero JM Almeida J Marcos M Orfao A. Chronic alcohol consumption is associated with changes in the distribution, immunophenotype, and the inflammatory cytokine secretion profile of circulating dendritic cells. Alcohol Clin Exp Res. (2007) 31:846–54. 10.1111/j.1530-0277.2007.00377.x 17386065

[85] Buttari B Profumo E Mancinelli R Cesta Incani U Tosti ME Attilia ML. Chronic and acute alcohol exposure prevents monocyte-derived dendritic cells from differentiating and maturing. Int J Immunopathol Pharmacol. (2008) 21:929–39. 10.1177/039463200802100417 19144278

[86] Heinz R Waltenbaugh C. Ethanol consumption modifies dendritic cell antigen presentation in mice. Alcohol Clin Exp Res. (2007) 31:1759–71. 10.1111/j.1530-0277.2007.00479.x 17850646

[87] Lau AH Abe M Thomson AW. Ethanol affects the generation, cosignaling molecule expression, and function of plasmacytoid and myeloid dendritic cell subsets *in vitro* and *in vivo*. J Leukoc Biol. (2006) 79:941–53. 10.1189/jlb.0905517 16478920

[88] Fan J Edsen-Moore MR Turner LE Cook RT Legge KL Waldschmidt TJ. Mechanisms by which chronic ethanol feeding limits the ability of dendritic cells to stimulate T-cell proliferation. Alcohol Clin Exp Res. (2011) 35:47–59. 10.1111/j.1530-0277.2010.01321.x 21039629 PMC3058243

[89] Siggins RW Bagby GJ Molina P Dufour J Nelson S Zhang P. Alcohol exposure impairs myeloid dendritic cell function in rhesus macaques. Alcohol Clin Exp Res. (2009) 33:1524–31. 10.1111/j.1530-0277.2009.00980.x 19485975 PMC2948236

[90] Agudelo M Figueroa G Parira T Yndart A Muñoz K Atluri V. Profile of class I histone deacetylases (HDAC) by human dendritic cells after alcohol consumption and *in vitro* alcohol treatment and their implication in oxidative stress: role of HDAC inhibitors trichostatin A and mocetinostat. PLoS ONE. (2016) 11:e0156421. 10.1371/journal.pone.0156421 27249803 PMC4889108

[91] Parira T Figueroa G Laverde A Casteleiro G Gomez Hernandez ME Fernandez-Lima F Novel detection of post-translational modifications in human monocyte-derived dendritic cells after chronic alcohol exposure: role of inflammation regulator H4K12ac. Sci Rep. (2017) 7:11236 10.1038/s41598-017-11172-6 28894190 PMC5593989

[92] Boukli NM Saiyed ZM Ricaurte M Rodriguez JW Ríos Olivares E Cubano LA. Implications of ER stress, the unfolded protein response, and pro- and anti-apoptotic protein fingerprints in human monocyte-derived dendritic cells treated with alcohol. Alcohol Clin Exp Res. (2010) 34:2081–8. 10.1111/j.1530-0277.2010.01304.x 20860616 PMC2988982

[93] Albano E. Role of adaptive immunity in alcoholic liver disease. Int J Hepatol. (2012) 2012:893026. 10.1155/2012/893026 22229098 PMC3168273

[94] Pasala S Barr T Messaoudi I. Impact of alcohol abuse on the adaptive immune system. Alcohol Res. (2015) 37:185–97. 26695744 10.35946/arcr.v37.2.04 PMC4590616

[95] Cho YE Im EJ Moon PG Mezey E Song BJ Baek MC. Increased liver-specific proteins in circulating extracellular vesicles as potential biomarkers for drug- and alcohol-induced liver injury. PLoS ONE. (2017) 12:e0172463. 10.1371/journal.pone.0172463 28225807 PMC5321292

[96] Li S Hong M Tan HY Wang N Feng Y. Insights into the role and interdependence of oxidative stress and inflammation in liver diseases. Oxid Med Cell Longev. (2016) 2016:4234061. 10.1155/2016/4234061 28070230 PMC5192343

[97] Galicia-Moreno M Rosique-Oramas D Medina-Avila Z Álvarez-Torres T Falcón D Higuera-de la Tijera F. Behavior of oxidative stress markers in alcoholic liver cirrhosis patients. Oxid Med Cell Longev. (2016) 2016:9370565. 10.1155/2016/9370565 28074118 PMC5198187

[98] Vidali M Stewart SF Albano E. Interplay between oxidative stress and immunity in the progression of alcohol-mediated liver injury. Trends Mol Med. (2008) 14:63–71. 10.1016/j.molmed.2007.12.005 18222109

[99] Liu YK. Leukopenia in alcoholics. Am J Med. (1973) 54:605–10. 10.1016/0002-9343(73)90118-6 4701945

[100] Mcfarland W Libre EP. Abnormal Leukocyte Response in Alcoholism. Ann Intern Med. (1963) 59:865–77. 10.7326/0003-4819-59-6-865 14082738

[101] Matos LC Batista P Monteiro N Ribeiro J Cipriano MA Henriques P. Lymphocyte subsets in alcoholic liver disease. World J Hepatol. (2013) 5:46–55. 10.4254/wjh.v5.i2.46 23646229 PMC3642723

[102] Skolarus TA Metreger T Wittmann D Hwang S Kim HM Grubb RL. Self-management in long-term prostate cancer survivors: a randomized, controlled trial. J Clin Oncol. (2019) 37:1326–35. 10.1200/JCO.18.01770 30925126 PMC6524986

[103] Almeida J Polvorosa MA Gonzalez-Quintela A Marcos M Pastor I Hernandez Cerceño ML. Decreased peripheral blood CD4+/CD25+ regulatory T cells in patients with alcoholic hepatitis. Alcohol Clin Exp Res. (2013) 37:1361–9. 10.1111/acer.12095 23550693

[104] Ortiz V Wands JR. Chronic ethanol diet increases regulatory T-cell activity and inhibits hepatitis C virus core-specific cellular immune responses in mice. Hepatol Res. (2014) 44:788–97. 10.1111/hepr.12173 23710581 PMC3883867

[105] Hammerich L Heymann F Tacke F. Role of IL-17 and Th17 cells in liver diseases. Clin Dev Immunol. (2011) 2011:345803. 10.1155/2011/345803 21197451 PMC3010664

[106] Lemmers A Moreno C Gustot T Maréchal R Degré D Demetter P. The interleukin-17 pathway is involved in human alcoholic liver disease. Hepatology. (2009) 49:646–57. 10.1002/hep.22680 19177575

[107] Xiao X Cai J. Mucosal-Associated Invariant T Cells: New insights into antigen recognition and activation. Front Immunol. (2017). 8:1540. 10.3389/fimmu.2017.01540 29176983 PMC5686390

[108] Riva A Patel V Kurioka A Jeffery HC Wright G Tarff S. Mucosa-associated invariant T cells link intestinal immunity with antibacterial immune defects in alcoholic liver disease. Gut. (2018) 67:918–30. 10.1136/gutjnl-2017-314458 29097439 PMC5890654

[109] Gao B Ma J Xiang X. MAIT cells: a novel therapeutic target for alcoholic liver disease? Gut. (2018) 67:784–6. 10.1136/gutjnl-2017-315284 29167178

[110] Perney P Portalès P Clot J Blanc F Corbeau P. Diminished CD4+ T cell surface CCR5 expression in alcoholic patients. Alcohol Alcohol. (2004) 39:484–5. 10.1093/alcalc/agh096 15498817

[111] Chen RC Xu LM Du SJ Huang SS Wu H Dong JJ. Lactobacillus rhamnosus GG supernatant promotes intestinal barrier function, balances Treg and TH17 cells and ameliorates hepatic injury in a mouse model of chronic-binge alcohol feeding. Toxicol Lett. (2016) 241:103–10. 10.1016/j.toxlet.2015.11.019 26617183

[112] Song K Coleman RA Alber C Ballas ZK Waldschmidt TJ Mortari F. TH1 cytokine response of CD57+ T-cell subsets in healthy controls and patients with alcoholic liver disease. Alcohol. (2001) 24:155–67. 10.1016/S0741-8329(01)00146-X 11557301

[113] Støy S Sandahl TD Dige AK Agnholt J Rasmussen TK Grønbæk H. Highest frequencies of interleukin-22-producing T helper cells in alcoholic hepatitis patients with a favourable short-term course. PLoS ONE. (2013) 8:e55101. 10.1371/journal.pone.0055101 23372820 PMC3555927

[114] Laso FJ Iglesias-Osma C Ciudad J López A Pastor I Torres E. Alcoholic liver cirrhosis is associated with a decreased expression of the CD28 costimulatory molecule, a lower ability of T cells to bind exogenous IL-2, and increased soluble CD8 levels. Cytometry. (2000) 42:290–5. 10.1002/1097-0320(20001015)42:5<290::AID-CYTO6>3.3.CO;2-X 11025487

[115] Lin F Taylor NJ Su H Huang X Hussain MJ Abeles RD. Alcohol dehydrogenase-specific T-cell responses are associated with alcohol consumption in patients with alcohol-related cirrhosis. Hepatology. (2013) 58:314–24. 10.1002/hep.26334 23424168

[116] Markwick LJ Riva A Ryan JM Cooksley H Palma E Tranah TH. Blockade of PD1 and TIM3 restores innate and adaptive immunity in patients with acute alcoholic hepatitis. Gastroenterology. (2015) 148:590–602 e10. 10.1053/j.gastro.2014.11.041 25479137

[117] Parra D Takizawa F Sunyer JO. Evolution of B cell immunity. Annu Rev Anim Biosci. (2013) 1:65–97. 10.1146/annurev-animal-031412-103651 25340015 PMC4203447

[118] Zhang H Meadows GG. Chronic alcohol consumption in mice increases the proportion of peripheral memory T cells by homeostatic proliferation. J Leukoc Biol. (2005) 78:1070–80. 10.1189/jlb.0605317 16260584

[119] Wang H Zhou H Mahler S Chervenak R Wolcott M. Alcohol affects the late differentiation of progenitor B cells. Alcohol Alcohol. (2011) 46:26–32. 10.1093/alcalc/agq076 21098503 PMC3002845

[120] Massonnet B Delwail A Ayrault JM Chagneau-Derrode C Lecron JC Silvain C. Increased immunoglobulin A in alcoholic liver cirrhosis: exploring the response of B cells to Toll-like receptor 9 activation. Clin Exp Immunol. (2009) 158:115–24. 10.1111/j.1365-2249.2009.04004.x 19737238 PMC2759066

[121] Andrade MC Albernaz MJ Araújo MS Santos BP Teixeira-Carvalho A Faria AM. Short-term administration of ethanol in mice deviates antigen presentation activity towards B cells. Scand J Immunol. (2009) 70:226–37. 10.1111/j.1365-3083.2009.02289.x 19703012

[122] Zeng T Zhang CL Xiao M Yang R Xie KQ. Critical roles of kupffer cells in the pathogenesis of alcoholic liver disease: from basic science to clinical trials. Front Immunol. (2016) 7:538. 10.3389/fimmu.2016.00538 27965666 PMC5126119

[123] Liu P Chen L Zhang H. Natural killer cells in liver disease and hepatocellular carcinoma and the NK cell-based immunotherapy. J Immunol Res. (2018) 2018:1206737. 10.1155/2018/1206737 30255103 PMC6142725

[124] Jeong WI Gao B. Innate immunity and alcoholic liver fibrosis. J Gastroenterol Hepatol. (2008) 23(Suppl. 1):S112–8. 10.1111/j.1440-1746.2007.05274.x 18336653 PMC2737176

[125] Melhem A Muhanna N Bishara A Alvarez CE Ilan Y Bishara T. Anti-fibrotic activity of NK cells in experimental liver injury through killing of activated HSC. J Hepatol. (2006) 45:60–71. 10.1016/j.jhep.2005.12.025 16515819

[126] Kim MJ Nagy LE Park PH. Globular adiponectin inhibits ethanol-induced reactive oxygen species production through modulation of NADPH oxidase in macrophages: involvement of liver kinase B1/AMP-activated protein kinase pathway. Mol Pharmacol. (2014) 86:284–96. 10.1124/mol.114.093039 24850909 PMC6067636

[127] Wang M Shen G Xu L Liu X Brown JM Feng D IL-1 receptor like 1 protects against alcoholic liver injury by limiting NF-κB activation in hepatic macrophages. J Hepatol. (2017) 68:109–17. 10.1016/j.jhep.2017.08.023 28870670

[128] McCullough RL McMullen MR Poulsen KL Kim A Medof ME Nagy LE. Anaphylatoxin Receptors C3aR and C5aR1 Are Important Factors That Influence the Impact of Ethanol on the Adipose Secretome. Front Immunol. (2018) 9:2133. 10.3389/fimmu.2018.02133 30294325 PMC6158367

[129] Motomura K Ohata M Satre M Tsukamoto H. Destabilization of TNF-alpha mRNA by retinoic acid in hepatic macrophages: implications for alcoholic liver disease. Am J Physiol Endocrinol Metab. (2001) 281:E420–9. 10.1152/ajpendo.2001.281.3.E420 11500296

[130] Chen Y Ouyang X Hoque R Garcia-Martinez I Yousaf MN Tonack S. beta-Hydroxybutyrate protects from alcohol-induced liver injury via a Hcar2-cAMP dependent pathway. J Hepatol. (2018) 69:687–96. 10.1016/j.jhep.2018.04.004 29705237 PMC6098974

[131] Lu KH Liu CT Raghu R Sheen LY. Therapeutic potential of chinese herbal medicines in alcoholic liver disease. J Tradit Complement Med. (2012) 2:115–22. 10.1016/S2225-4110(16)30084-0 24716123 PMC3942913

[132] Fu X Zhong Z Hu F Zhang Y Li C Yan P. The protective effects of selenium-enriched Spirulina platensis on chronic alcohol-induced liver injury in mice. Food Funct. (2018) 9:3155–65. 10.1039/C8FO00477C 29862408

[133] Kim HK Kim DS Cho HY. Protective effects of Platycodi radix on alcohol-induced fatty liver. Biosci Biotechnol Biochem. (2007) 71:1550–2. 10.1271/bbb.60523 17587688

[134] Khanal T Choi JH Hwang YP Chung YC Jeong HG. Protective effects of saponins from the root of Platycodon grandiflorum against fatty liver in chronic ethanol feeding via the activation of AMP-dependent protein kinase. Food Chem Toxicol. (2009) 47:2749–54. 10.1016/j.fct.2009.08.006 19683027

[135] Khanal T Choi JH Hwang YP Chung YC Jeong HG. Saponins isolated from the root of Platycodon grandiflorum protect against acute ethanol-induced hepatotoxicity in mice. Food Chem Toxicol. (2009) 47:530–5. 10.1016/j.fct.2008.12.009 19133309

[136] Feng Y Cheung KF Wang N Liu P Nagamatsu T Tong Y. Chinese medicines as a resource for liver fibrosis treatment. Chin Med. (2009) 4:16. 10.1186/1749-8546-4-16 19695098 PMC3224967

[137] Dhiman RK Chawla YK. Herbal medicines for liver diseases. Dig Dis Sci. (2005) 50:1807–12. 10.1007/s10620-005-2942-9 16187178

[138] Kaviarasan S Ramamurty N Gunasekaran P Varalakshmi E Anuradha CV. Fenugreek (Trigonella foenum graecum) seed extract prevents ethanol-induced toxicity and apoptosis in Chang liver cells. Alcohol Alcohol. (2006) 41:267–73. 10.1093/alcalc/agl020 16574673

[139] Balaban YH Aka C Koca-Caliskan U. Liver immunology and herbal treatment. World J Hepatol. (2017) 9:757–70. 10.4254/wjh.v9.i17.757 28660010 PMC5474722

[140] Vargas-Mendoza N Madrigal-Santillán E Morales-González A Esquivel-Soto J Esquivel-Chirino C García-Luna Y González-Rubio M. Hepatoprotective effect of silymarin. World J Hepatol. (2014) 6:144–9. 24672644 10.4254/wjh.v6.i3.144 PMC3959115

[141] Tamayo C Diamond S. Review of clinical trials evaluating safety and efficacy of milk thistle (*Silybum marianum [L.] Gaertn*.). Integr Cancer Ther. (2007) 6:146–57. 10.1177/1534735407301942 17548793

